# Numerical Study of Plasmonic Efficiency of Gold Nanostripes for Molecule Detection

**DOI:** 10.1155/2015/724123

**Published:** 2015-02-03

**Authors:** Thomas Grosges, Dominique Barchiesi

**Affiliations:** Group for Automatic Mesh Generation and Advanced Methods, Gamma3 (UTT-INRIA), University of Technology of Troyes, 12 rue Marie Curie-CS 42060, 10004 Troyes Cedex, France

## Abstract

In plasmonics, the accurate computation of the electromagnetic field enhancement is necessary in determining the amplitude and the spatial extension of the field around nanostructures. Here, the problem of the interaction between an electromagnetic excitation and gold nanostripes is solved. An optimization scheme, including an adaptive remeshing process with error estimator, is used to solve the problem through a finite element method. The variations of the electromagnetic field amplitude and the plasmonic active zones around nanostructures for molecule detection are studied in this paper taking into account the physical and geometrical parameters of the nanostripes. The evolution between the sizes and number of nanostripes is shown.

## 1. Introduction

In the past decades, scientists have been interested in the origin and the mechanism of electric field enhancement around nanostructures, in particular for surface enhanced Raman scattering (SERS) [1, 2] and their applications in molecule detection [3, 4]. The main part of the field enhancement arises from the amplification of the electric field near metallic surfaces and involves the excitation of the localised surface plasmon resonances. In SERS process, the nanoparticles can play the role of nanoantennas for the molecule and the particles enhance the incoming electromagnetic fields both at the frequency of illumination and at the Raman shifted frequency [5].

To enhance the SERS signal, a great variety of nanostructured substrates have been used such as metal islands films [6], nanospheres lithography [7], or the so-called “natural lithography” techniques, that uses of anodic nanoporous alumina resulting from aluminum anodization [8], and arrays of lithographically designed particles [3, 9, 10]. To study the influence of localized plasmon resonances and field enhancement factor, regular arrays of identical metallic nanoparticles and metallic nanostripes obtained by electron beam lithography are widely used [5, 10, 11]. The ability of these systems is the narrow localized surface plasmon resonances and their tunability (through the change of the particle's material, size, and shape). Moreover, in such structures and in case of high quality fabricated patterns which can be difficult to obtain and that are time consuming and demanding in terms of facility requirements, the Raman enhancement factor is distributed almost equally over all particles [12, 13].

In this context, a numerical model, allowing computing with accuracy the electric field enhancement around gold nanostripes, is presented. The spatial evolution of the field, associated with the number of gold nanostripes is studied. The numerical optimization, including the adaptive remeshing scheme with error estimator based on the Hessian of the solution, takes into account the variations of the field enhancement and ensures the convergence of the solution to the physical solution [14, 15]. The paper is organized as follows: Section 2 presents the equations of the model, the numerical resolution method, the adaptive remeshing scheme, and the optimization steps. In Section 3, the results of numerical simulations are presented before concluding.

## 2. Model, Numerical Method, and Optimization

This section presents the numerical method used to solve the electromagnetic problem.

### 2.1. Finite Element Method Applied to the Electromagnetic Problem

The objective is to solve the wave (or Helmoltz) equations for the system with complex geometries and to find the electromagnetic field. The finite element method (FEM) was applied for many years in mechanics, thermodynamics, electromagnetics, and electrical engineering [16, 17] and consists in solving systems of partial differential equation with boundary conditions in open or closed domains. The general problem is solved on a discrete mesh of the domain [18] and the electromagnetic fields are computed at the nodes of the mesh by using a variational method. To control the error on the solution and to limit the increase of the number of nodes and of the computational time, an improved method, including a process of iterative remeshing, is proposed and applied. Such an optimized FEM allows describing complex structures including arbitrary geometries and shapes [18–20]. The use of a weak formulation (or variational formulation) for the electromagnetic equations also improves the stability of the FEM. For 2D case (i.e., infinite geometry along the *z*-axis), a weak formulation is used for the Helmoltz partial differential equation of the magnetic field *H*_*z*_ for a polarized illumination in the transverse magnetic mode TM. The magnetic field is reduced to a scalar problem and satisfies

(1) ∫Ω1ϵr∇Hzx,y·∇ψ(x,y)−k02Hz(x,y)ψ(x,y)dΩ =0,

where *k*_0_ = *ω*/*c* is the wave number of the monochromatic incoming wave of angular frequency *ω*, *c* is the velocity of ligth in vacuum, and *ϵ*_*r*_ is the relative complex permittivity of the materials that are functions of the spatial coordinates (*x*, *y*). The test function *ψ* is defined on *L* ^2^(*Ω*) (i.e., the linear space of the scalar functions *ψ* that is square-integrable on *Ω*). Such a basis of polynomial functions gives an approximation of the solution *H*_*z*_ in each element of the mesh [21]. The field *H*_*z*_ is a linear combination of basic polynomial functions *ψ* (e.g., P2 polynomial functions of degree 2 in order to ensure a nonconstant derivated electric field) and the problem is reduced to solve a linear system [17, 22]. With the given boundary conditions, the partial differential equation is exactly verified at each node of the mesh by the solution. Here, the Ritz's formulation of the variational problem is implemented to automatically satisfy the continuity of the tangential components of the electromagnetic field [18]. The electric field amplitude is deduced from the Maxwell-Ampère equation [23] and is given by

(2) Ex,y =1k0cϵrϵ0  ·∂xHz(x,y)∂xHz∗(x,y)+∂yHz(x,y)∂yHz∗(x,y)1/2,

where ∗ denotes the complex conjugate of the *H*_*z*_-field, *ϵ*_0_ is the permittivity of vacuum, and ∂*x*, ∂*x* are the derivative operators along *x*- and *y*-axes, respectively.

### 2.2. Adaptive Remeshing and Optimization Scheme

The partial differential equation is solved on a mesh of the computational domain through the FEM. The accuracy of the computed solution is closely related to the quality of the mesh [15, 24, 25]. The improvement of the quality of the solutions by adapting the size of the mesh elements to the physical solution [15, 26] is implemented through the remeshing process with adaptive loops. In plasmonics systems, where strong variations of the electromagnetic field occur, the convergence of the solution to a stable solution requires mesh adaptions. At each step of the adaption process, the approximations of the solutions of the Helmholtz equation *H*_*z*_, the electric field **E**, and electric field amplitude |**E**| are calculated [26]. The maximum deviation between the solution associated with the mesh and the exact solution is limited by using the interpolation error (which is based on an estimation of the discrete Hessian of the solution) [27, 28]. From the interpolation error, an* a posteriori* error estimator allows defining a physical size map *S*_*p*_(*Ω*) such as

(3) $$\begin{array}{c} S_{p}\left(\Omega \right)=\left\{h_{p}\left(x,y\right)\right\},\;\forall \left(x,y\right)\in \Omega , \end{array}$$

where *h*_*p*_(*x*, *y*) is the physical size defined at each node of the mesh. This physical size is proportional to the inverse of the deviation of the Hessian. For a given maximum tolerance *β*, the physical size *h*_*p*_(*x*, *y*) is given by

(4) $$\begin{array}{c} h_{\min}\leq h_{p}\left(x,y\right)=\frac{\beta }{\eta \left(x,y\right)}\leq h_{\max}, \end{array}$$

where *η*(*x*, *y*) estimates the maximum deviation and is obtained from the Hessian of the solution and the minimum and maximum sizes of the elements are *h*_min⁡_ and *h*_max⁡_, respectively. This size map *S*_*p*_(*Ω*) is obtained from BL2D-V2 software (adaptive remeshing generating isotropic or anisotropic meshes) [29], to govern the remeshing of the domain. Therefore, the domain is entirely remeshed and a new mesh *M*_*p*_(*Ω*) is constructed. That contrasts with basic remeshing methods that only add nodes in the mesh of the previous step of remeshing. The resolution of the plasmonic problem is based on the computation of the physical size map *S*_|**E**|_(*Ω*) related to the amplitude of the electric field **E**. The optimized computational scheme consists in iterative and adaptive loops:

1. construction of the initial mesh *M*_*i*=0_(*Ω*) with triangular elements in the computational domain *Ω*,
2. computation of the magnetic field (*H*_*z*_)_*i*_ (i.e., solution of (1)) on *M*_*i*_(*Ω*),
3. derivation of the electric field **E**_*i*_ and computation of the amplitude (i.e., solution of (2)) on *M*_*i*_(*Ω*),
4. estimation of the physical error: computation of the interpolation error on the physical solution |**E**_*i*_|; definition of a physical size map *S*_|**E**_*i*_|_(*Ω*) connected to the field amplitude |**E**_*i*_| enabling to relate the error to a given threshold *β* = *δ*_|**E**|_,
5. remeshing of the domain conforming to the size map *S*_|**E**_*i*_|_(*Ω*),
6. if the threshold  *δ*_|**E**|_ is not reached: loop to step (2), with *i* = *i* + 1, in order to obtain a new mesh *M*_*i*_(*Ω*), else *M*_|**E**|_(*Ω*) = *M*_*i*_(*Ω*).

Due to the optimization of the position of the new vertex in respect to the* a posteriori* interpolation error achieved on the **E**-field, the adaptive remeshing procedure permits reducing the number of the iterations and controling the accuracy of the solution. This also contrasts the basic adaptive process where two loop sequences are necessary: the first one on the error on the PDE solution (*β* = *δ*_*H*_) and the second one on the error on the **E**-field (*β* = *δ*_|**E**|_) [26].

## 3. Numerical Results and Discussion

**Figure 1:**
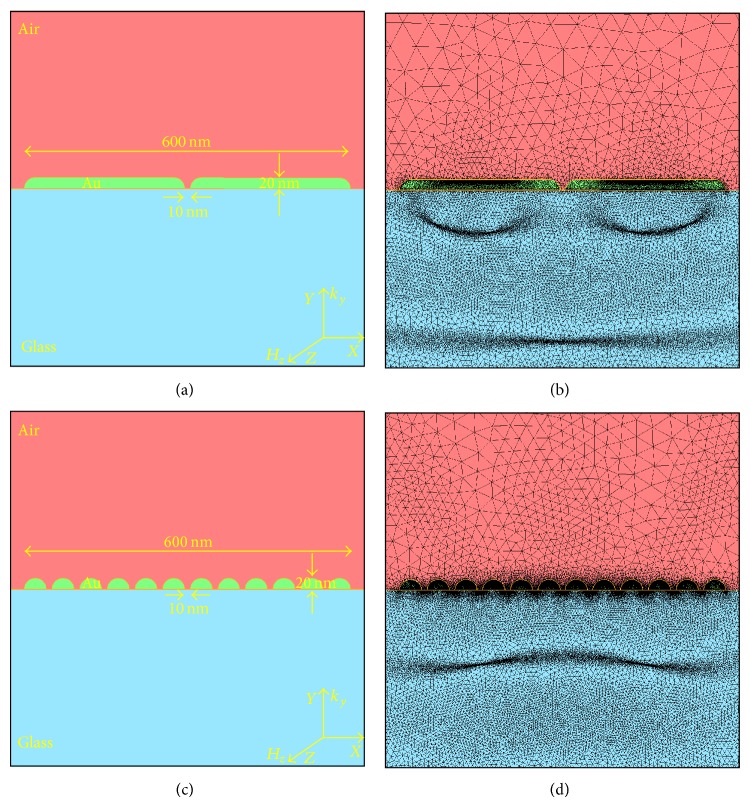
Geometries ((a), (c)) and the final adapted meshes ((b), (d)) for 2 and 12 gold nanostripes, respectively.

Here, we consider nanostripes of total width *L* = 600 nm and height *b* = 20 nm deposited on glass plate (see Figure 1).

These gold lines are subdivided into *N*_stripe_ gold nanostripes (with 2 ≤ *N*_stripe_ ≤ 12), each nanostripe being separated by a gap width *l*_gap⁡_ = 10 nm. Therefore the total length of the gold structure is *L* = *a* · *N*_stripe_ + *l*_gap⁡_(*N*_stripe_ − 1) and the width of each gold nanostripe is *a* = [*L* − *l*_gap⁡_(*N*_stripe_ − 1)]/*N*_stripe_. Figures 1(a)–1(d) illustrate the geometries of the system and the associated meshes for 2 and 12 gold nanostripes, respectively. The relative permittivities are *ϵ*_*r*_(Air) = 1.00, *ϵ*_*r*_(Glass) = 2.25, and *ϵ*_*r*_(Au) = −11.75 + *j*1.25; the sample is illuminated by a TM polarized excitation at wavelength *λ* = 632 nm on normal incidence in glass (see Figure 1). The materials of the system are considered isotropic and homogeneous.

**Figure 2:**
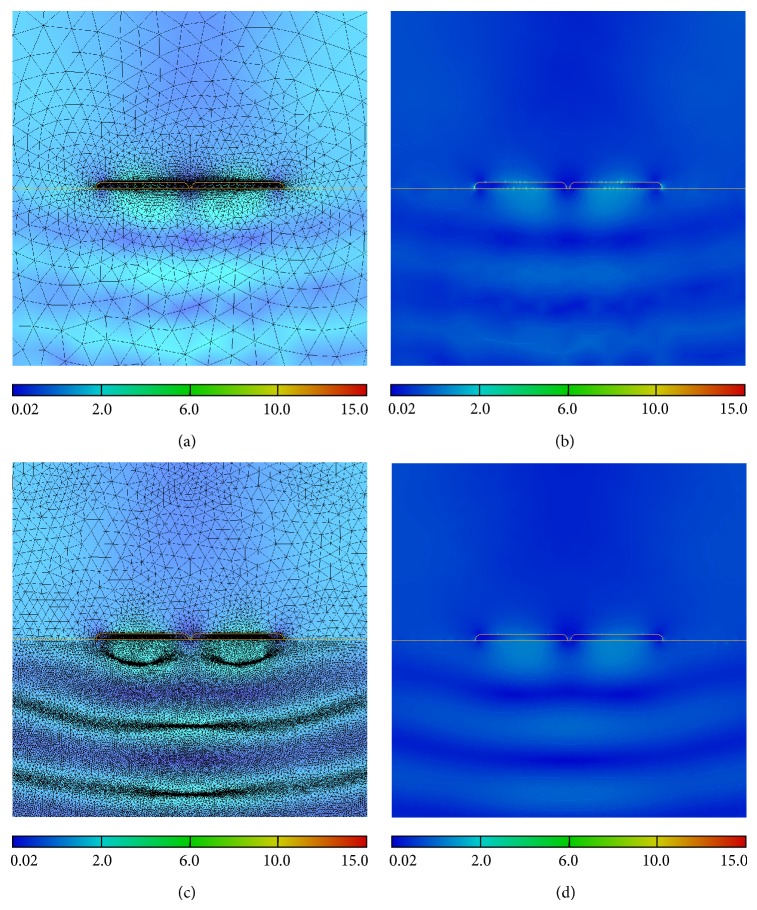
Electric field amplitude for 2 gold nanostripes for the initial (a) mesh and (b) computation and after adaption of the (c) mesh and (d) the field computation, respectively.

The results of the adaptive process on meshes and on the electric field maps are illustrated on Figure 2. Figures 2(a)-2(b) show the initial mesh *M*_0_ and the associated electric field amplitude for the *N*_stripe_ = 2 gold nanostripes. The adaptive process on the electric field amplitude |**E**| (with *h*_max⁡_ = 200 nm, *h*_min⁡_ = 0.01 nm, and *δ*_|**E**|_ = 0.001) produces the adapted mesh *M*_|**E**|_ and the field amplitude map (Figures 2(c)-2(d)). The mesh is adapted where the field presents strong variations of amplitude. The final mesh *M*_|**E**|_ is obtained, after five iterations, by applying the adaptive process on the field **E** (with *δ*_|**E**|_ = 0.001). The remeshing process takes into account not only the shape and size of the nanostripes but also the total field variations. Moreover, we can remark that the final adapted meshes, Figure 2(c), include not only the local field enhancements but also the effects of the reflected waves. We can also mention the drastic reduction of the artifactual enhancement of **E**-field near the surfaces in Figure 2(b) after the remeshing process (see Figure 2(d)).

**Figure 3:**
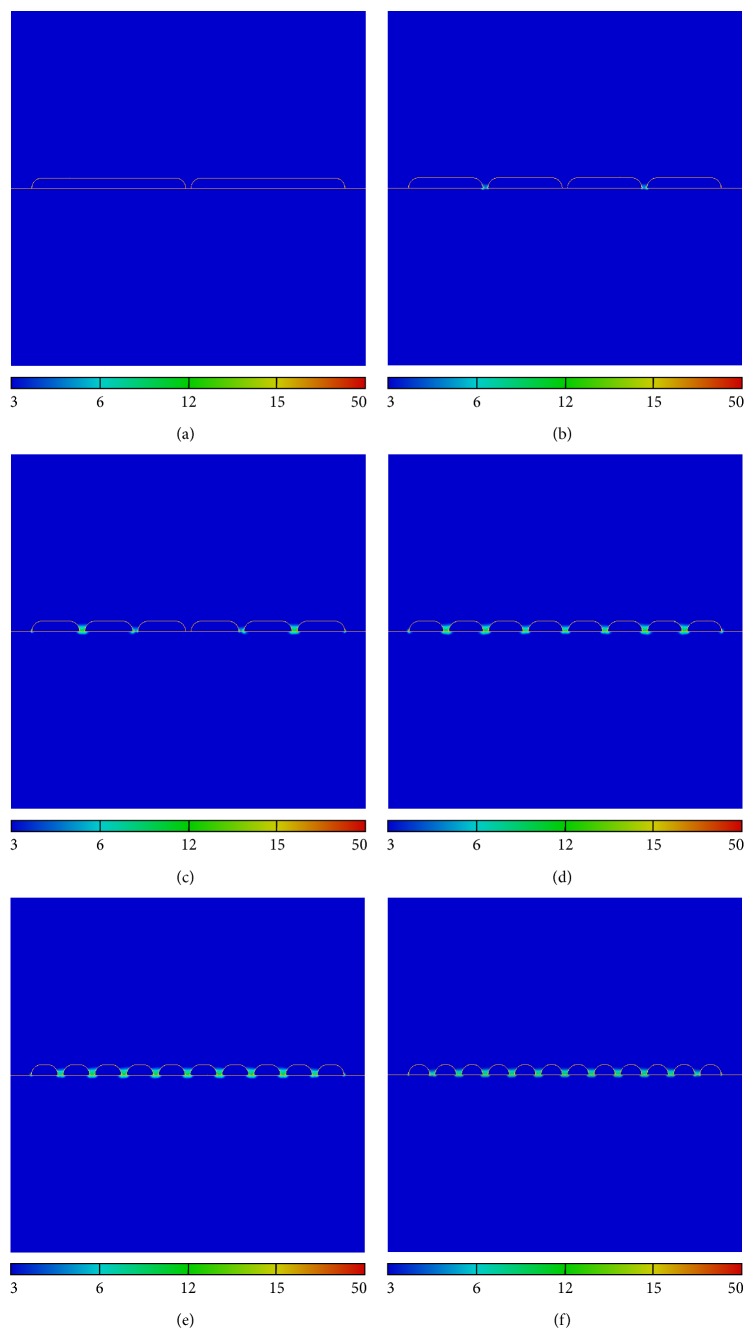
Spatial maps of the electric field amplitude for (a) 2, (b) 4, (c) 6, (d) 8, (e) 10, and (f) 12 gold nanostripes illuminated at *λ* = 632 nm.

**Figure 4:**
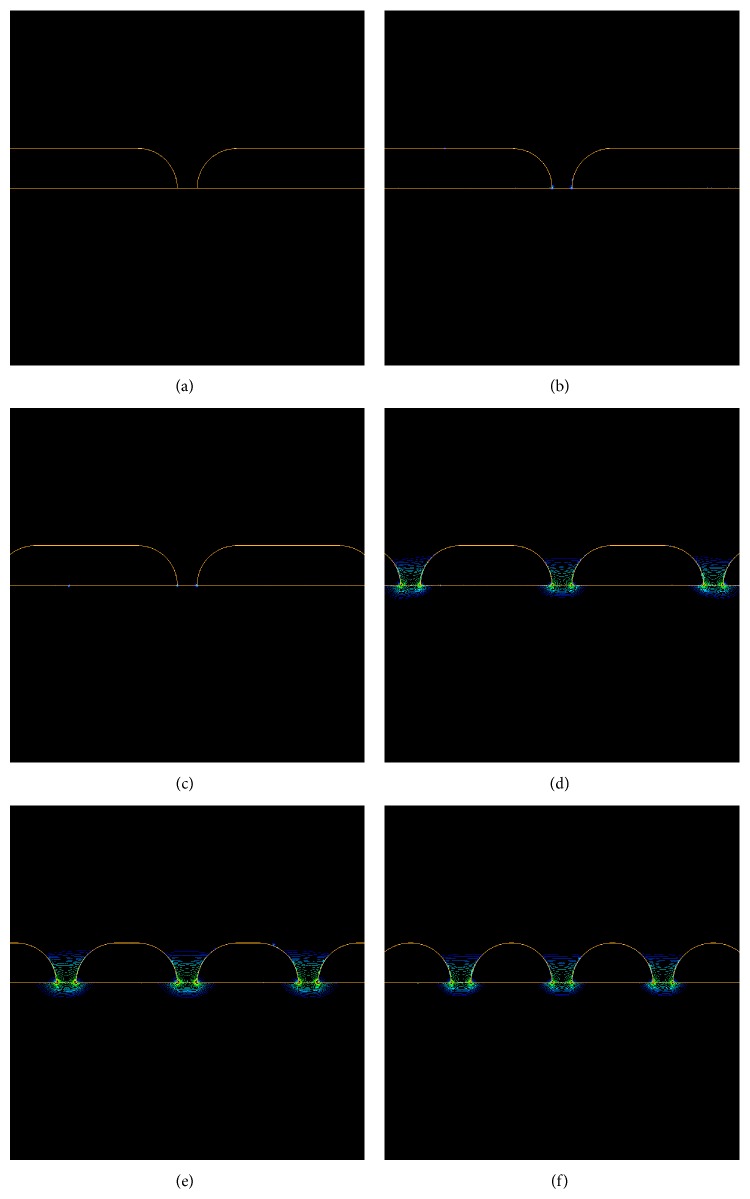
Zoom in the gap zone on spatial contours of the electric field for (a) 2, (b) 4, (c) 6, (d) 8, (e) 10, and (f) 12 gold nanostripes illuminated at *λ* = 632 nm. The amplitudes and spatial extensions are shown.

In order to study the evolution of the electric field amplitude and the efficiency of the plasmonic system, for a given total length *L* of the structure, we also consider various numbers of gold nanostripes *N*_stripe_ (or number of gaps *N*_gap⁡_ = *N*_stripe_ − 1). Figures 3(a)–3(f) show the amplitude maps of the electric field for *N*_stripe_ = 2, 4, 6, 8, 10, and 12 gold nanostripes, respectively. The evolution of the electric amplitude as function of the number of gold nanostripes can be compared. The zoom on the contours (see Figure 4) permits showing and identifying, for a given amplitude threshold, the location of the active zone of detection (i.e., where the plasmonic effects are located and their spatial extension). The main result is, for *N*_stripe_ increasing, the electric field amplitude and the active plasmonic zone increase to a maximum before decreasing.

**Figure 5:**
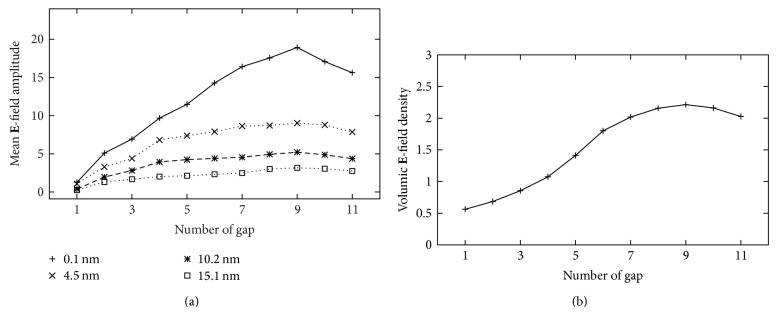
Evolution of (a) the mean electric amplitude as function of the number of gap and for four distance from the glass surface. Evolution of (b) the integrated electric amplitude density per volume as function of the number of gap. A maximum efficiency is reached at nine gaps (i.e., for ten nanostripes).

The theoretical efficiency of the detection can be reached by analysing the evolution of the mean electric amplitude as a function of the distance of the glass plate and the number of gold nanostripes *N*_stripe_ (or number of gaps *N*_gap⁡_ = *N*_stripe_ − 1).  Figure 5(a) shows a maximum of efficiency of the mean electric amplitude for a gold line subdivided in *N*_stripe_ = 10 (i.e., *N*_gap⁡_ = 9) nanostripes. The maximum amplitude of the electric field is reached near the glass plate in the gap between the nanostripes. Similarly, the structure presents a maximum in the integrated electric amplitude density per volume for *N*_stripe_ = 10 (i.e., for *N*_gap⁡_ = 9, see Figure 5(b)), where the volume of integration is *L* × *b*. That shows the efficiency of gold multinanostripes as field enhancement support, for biosensor applications where molecules are deposited on the whole surface of the biosensor (here in dry mode). In this case, the efficiency of the biosensor is directly proportional to the integrated electric amplitude.

Indeed, the efficiency of a biosensor is proportional to the size of the zone where the electric field is enhanced (which is related to the volumic field density). This property also can be characterized by the mean **E**-field amplitude (rather than the maximum of the amplitude value) that can be related to the level of signal that is expected in operating mode, when molecules of interest are spread near the sensor surface.

## 4. Conclusion

The paper focuses on the numerical optimization and simulation of gold stripes used in molecule detection. The adaption of the gap number between gold nanostripes is achieved in order to optimize both the electric field amplitude and the active zones of detection for a given threshold. That solution is computed by developing an adaptive remeshing method to compute with accuracy the electric field amplitude around gold nanostripes and by adapting the mesh to the evolution of the field, the material, and the geometry. The problem is solved through an adaptive loop process converging to a stable solution and decreasing the node numbers, the computation time, and the memory requirement. The influence of the gap number between gold nanostripes related to the electric field amplitude and the volumic field density is presented. The same method could systematically be used to analyse the efficiency of experimental sensor using metallic nanostripes, embeded in various media (e.g., for wet mode). Further studies will be devoted to the study of the dependance of the observed maximum as a function of the total length *L*, the gap size *l*_gap⁡_, and the height *b*. The advantage of such an adaptive method lies in its applicability to various simulation problems and in the optimization of complex systems in engineering [30–33]. The use of complex models can extend the domain of its application [34–36], especially to spectroscopic studies, using adapted models of fitting of the optical properties [37].
